# Recent advances in cutaneous lymphoma—implications for current and future classifications

**DOI:** 10.1007/s00428-022-03421-5

**Published:** 2022-10-24

**Authors:** JR Goodlad, L Cerroni, SH Swerdlow

**Affiliations:** 1 Department of Pathology, NHS Greater Glasgow and Clyde, Level 3 Laboratory Medicine Building Queen Elizabeth University Hospital, 1345 Govan Rd, Glasgow, G51 4TF UK; 2 Department of Dermatology, Medical University of Graz, Graz, Austria; 3 Department of Pathology, University of Pittsburgh School of Medicine, Pittsburgh, PA USA

**Keywords:** Cutaneous, Lymphoma, Lymphoproliferative disorder, Classification

## Abstract

The Revised European-American Classification of mature lymphoid neoplasms published in 1994 and the 2001, 2008 and 2016 WHO classifications that followed, were the product of international collaboration and consensus amongst haematopathologists, geneticists, molecular scientists and clinicians. Primary cutaneous lymphomas were fully incorporated into this process following the publication of the WHO-EORTC classification of cutaneous lymphomas in 2005. The definition, diagnostic criteria and recommended studies for primary cutaneous lymphoma continue to be refined. The 2022 International Consensus Classification represents the most recent update and an overview of all the main entities presenting primarily in the skin, together with the major changes in classification, are summarized herein. Primary cutaneous marginal zone lymphoma is segregated from other extranodal marginal zone lymphomas of mucosa-associated lymphoid tissue (MALT lymphoma) and downgraded to a lymphoproliferative disorder in line with its markedly indolent behaviour. In addition, two subtypes are recognised, based largely but not exclusively on whether they are heavy chain class-switched or IgM positive. Similarly, in keeping with a trend to greater conservatism, primary cutaneous acral CD8 positive T cell lymphoma is now also classified as a lymphoproliferative disorder. In addition, significant new insights into the biology of primary cutaneous lymphoma have also recently been forthcoming and will be presented. These studies have enhanced our knowledge of genetic, epigenetic and transcriptional changes in this group of diseases. They not only identify potential targets for novel therapies, but also raise as yet unanswered questions as to how we categorise cutaneous lymphomas, particularly with respect to relationships with similar lymphomas at extracutaneous sites.

## Introduction

Classification of cutaneous lymphomas has been a special challenge with initially both a skin-specific classification from the EORTC published in 1997 and the 2001 WHO classification with limited skin-specific entities to choose from [1, 2]. However, following the consensus meeting for the 2006 WHO classification of skin tumours, a further consensus meeting was held that led to the WHO-EORTC consensus classification of cutaneous lymphomas [3]. Much but not all of this classification was included in the subsequent 2008 and 2016 WHO classifications. Based on further discussions at the Clinical Advisory Committee (CAC), the International Consensus Classification of lymphomas (2022ICC) has continued this evolution, with multiple skin-specific entities recognised including segregation of what has been known as primary cutaneous marginal zone lymphoma.

Accurate diagnosis of the cutaneous lymphomas is essential in view of the broad range of clinical behaviour they exhibit, including lesions that spontaneously regress, lymphoid proliferations that that persist but pursue an indolent course and rapidly progressing aggressive malignancies that are often fatal. For some entities, the pathological appearances do not accurately predict biology, and certain disease subtypes show considerable overlap in the pathological features they display. Thus, a heavy emphasis is placed on clinical features when making certain diagnoses. Moreover, lymphomas of cutaneous origin often differ in clinical behaviour from morphologically similar lymphomas more usually arising in lymph nodes but with the potential to secondarily involve skin. Compounding all of the above, the rarity of most entities and lack of suitable tissue for study has hindered molecular analysis of this group of neoplasms and limited our knowledge of their underlying biology, particularly with respect to genetic abnormalities important to initiation and progression of disease.

Despite these handicaps, our understanding of cutaneous lymphomas continues to progress, aided by collaborative studies of large cohorts and advances in technology that have led to a more precise analysis of genetic, epigenetic and transcriptional changes in this group of diseases. This review represents an update on recent advances in the field and includes a summary of discussions that took place during, and surrounding, the September, 2021 Clinical Advisory Committee (CAC) meeting with input from pathologists, haematologists, oncologists and scientists from around the world. The CAC was a joint initiative of the Society for Hematopathology and the European Association for Haematopathology, culminating in the recently published International Consensus Classification of lymphomas (2022ICC) and myeloid neoplasms [4, 5]. The following text focuses on primary cutaneous lymphomas and lymphoproliferative disorders, defined as cutaneous lymphoid neoplasms with no evidence of extracutaneous disease at presentation, but it should be remembered that many types of systemic lymphoma can present in the skin as part of disseminated disease. This includes several types of EBV-related lymphomas/lymphoproliferative disorders that are discussed in detail elsewhere in this volume. The review presents revisions in nomenclature and summarises significant findings in some of the most recent genomic studies. It is not intended to be a comprehensive review of all types of cutaneous lymphomas. A list of primary cutaneous lymphomas, with changes from the updated 4th edition of the WHO classification highlighted, is given in Table 1.

**Table 1:** 2022 International Consensus Classification: primary cutaneous lymphoma and lymphoproliferative disorders

Mature B cell neoplasms
Primary cutaneous marginal zone lymphoproliferative disorder*
Primary cutaneous follicle centre lymphoma
Primary cutaneous DLBCL, leg type
EBV-positive mucocutaneous ulcer*
Mature T and NK-cell neoplasms
Mycosis fungoides
Sezary sydrome
Primary cutaneous CD30-positive lymphoproliferative disorders
Lymphomatoid papulosis
Primary cutaneous anaplastic large cell lymphoma
Primary cutaneous CD4-positive small/medium CD4-positive lymphoproliferative disorder
Subcutaneous panniculitis-like T-cell lymphoma
Primary cutaneous gamma-delta T-cell lymphoma
Primary cutaneous acral CD8-positive T-cell lymphoproliferative disorder*
Primary cutaneous CD8-positive aggressive epidermotropic cytotoxic T-cell lymphoma
Hydroa vacciniforme lymphoproliferative disorder*
Classic
Systemic

^*^Changes from 2016 WHO classification

## Primary cutaneous B-cell lymphomas and lymphoproliferative disorders

### Primary cutaneous marginal zone LPD

The revised 4th edition of the WHO Classification of Tumours of Haematopoietic and Lymphoid Tissues (2017) did not differentiate between extranodal marginal zone lymphoma of mucosa-associated lymphoid tissue (ENMZL/MALT lymphoma) arising primarily in the skin and ENMZL at other sites, although some differences were described. Consistent with the original 1997 EORTC classification of cutaneous lymphomas and the 2005 WHO-EORTC classification, subsequently incorporated into the 2006 WHO classification of skin tumours, and retained in the most recent WHO/WHO-EORTC classifications, the 2022ICC endorses recognition of cutaneous marginal zone lymphoma as a distinct entity on the basis of differences in histology, genetic profile and clinical behaviour in the majority of cases from many other MALT lymphomas [2, 3, 5–7]. In addition, primary cutaneous marginal zone lymphoma exhibits remarkably indolent behaviour. In large series, extracutaneous dissemination is seen in only 4–8.5% of cases and disease specific survival 5-year survival has been quoted to exceed 99% without recourse to aggressive therapy [8, 9]. There is also significant pathological overlap with cutaneous lymphoid hyperplasia, leading some to propose that cutaneous marginal zone lymphoma is better regarded as a clonal chronic lymphoproliferative disorder, probably related to some type of antigenic stimulation, rather than an overt lymphoma [10]. In light of these findings, the 2022ICC further adopted that primary cutaneous marginal zone lymphoma should be down-graded to a lymphoproliferative disorder [7]. Although primary cutaneous follicle centre lymphoma also has an excellent prognosis, there is a slightly higher incidence of extracutaneous spread and lymphoma related death. It was therefore preferred to retain designation of this entity as a lymphoma.

**Fig. 1:**
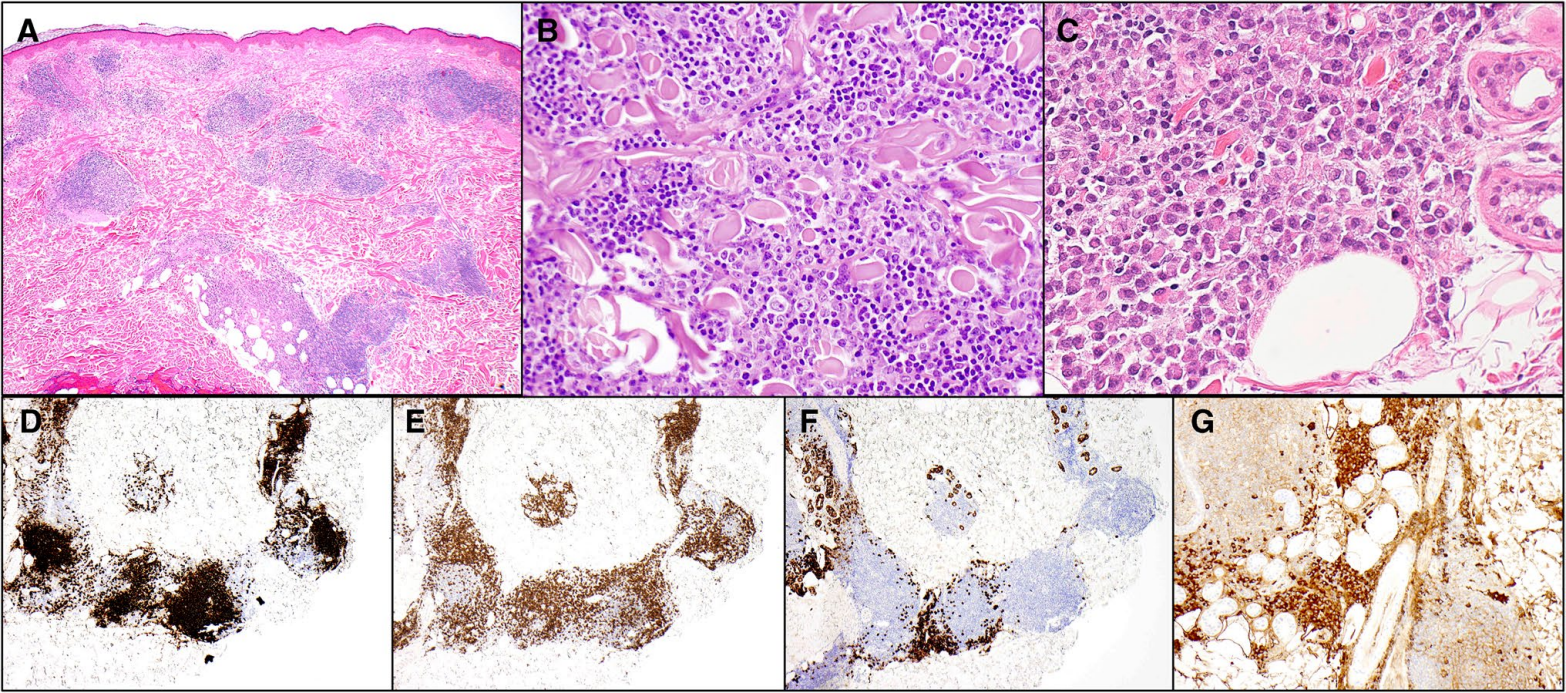
The majority of primary cutaneous marginal zone lymphoproliferative disorders display prominent plasmacytic differentiation. **A** Low magnification view of this example shows a nodular dermal infiltrate containing reactive lymphoid follicles. **B** The extrafollicular infiltrate contains a majority of small lymphocytes with scattered immunoblasts. **C** Aggregates of plasma cells are seen at the periphery of the infiltrate. **D** Staining for CD20 highlights B cell follicles, with few extrafollicular B-lymphocytes. **E** The majority of extrafollicular lymphocytes are reactive small T cells, highlighted by anti-CD3. **F** Anti-CD138 highlights aggregates of peripherally placed plasma cells. **G** The plasma cells are IgG positive, as they are in the majority of such lesions

Primary cutaneous marginal zone lymphoproliferative disorder (PCMZLPD) is a disease of adults and most patients present with solitary or clustered lesions in the form of erythematous papules, plaques or nodules. The trunk and upper extremities are most frequently involved [3]. Within this category, two types of primary cutaneous marginal zone lymphoproliferative disorder (PCMZLPD) can be recognised. The majority of PCMZLPD are class-switched, more frequently to IgG than IgA, and IgG4 positive in up to 40% of cases [11, 12]. Class switched PCMZLPD are typically centred in the dermis and contain a prominent background of reactive lymphoid tissue. Lymphoid follicles with germinal centres are common and lesions are T cell-rich with a Th2 microenvironment. Neoplastic cells are typically in a minority and show prominent plasmacytic differentiation (Fig. 1). The B cells generally do not express IRTA1 or CXCR3 [13–15]. Cutaneous recurrences are not infrequent but extracutaneous spread is exceptional.

**Fig. 2:**
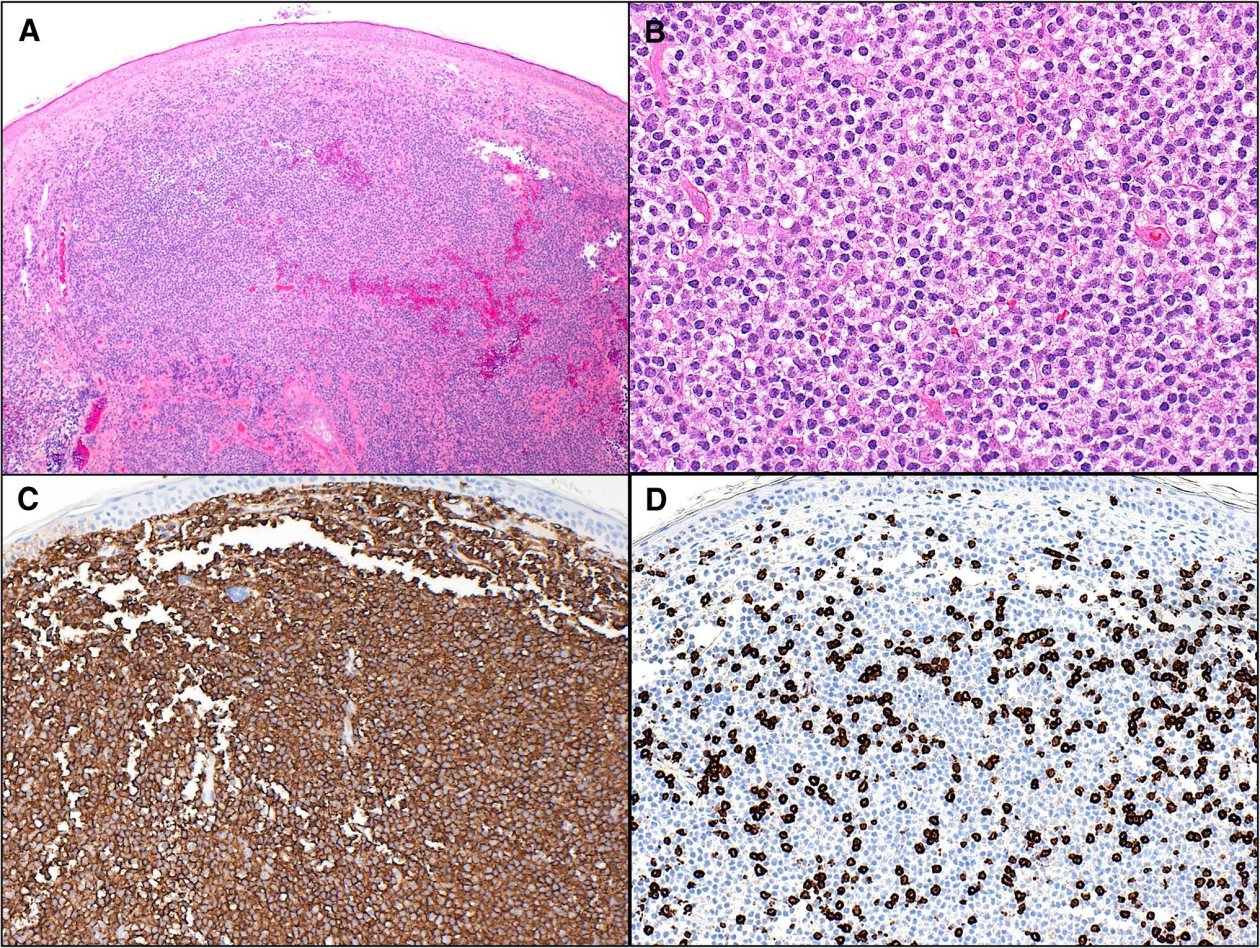
A subset of primary cutaneous marginal zone lymphoproliferative disorders are IgM positive. As in this example they more often show: **A** a diffuse pattern of growth and, **B** monocytoid morphology. **C** CD20: neoplastic B cells usually are in the majority and form confluent sheets. **D** CD3: Background reactive T cells are less conspicuous than in class switched cases

In contrast, IgM-positive PCMZLPD more often involves the subcutis and usually contains a predominance of neoplastic B cells that often express IRTA1 and CXCR3, and frequently display monocytoid or centrocyte-like morphology. Background reactive T cells are less conspicuous than in class switched cases and a Th1 microenvironment the norm (Fig. 2) [13–15]. Although rare, extracutaneous spread is more likely to be seen in this variant although prognosis remains excellent. Occasional patients with IgM^+^ PCMZL do have clonally-related class-switched PCMZLPDs that are IRTA1^+^ [13].

Chromosomal rearrangements have been reported in PCMZLPD, most frequently juxtaposing *MALT1* with IGH, but generally in only a minority of cases [16, 17]. The distinct nature of PCMZLPD is further highlighted by the report of mutations of the *FAS* gene in 63% of PCMZLPD, being identified in both IgM positive and class switched cases [18]. *MYD88* mutations are reported in a subset of IgM^+^ cases.

### Primary cutaneous follicle centre lymphoma

Primary cutaneous follicle centre lymphoma (PCFCL) typically presents with localised plaques, nodules or tumours, usually on the head and neck, particularly the scalp, or trunk. Other sites, including the legs, are less commonly the primary site of disease. Recommended treatment is local radiotherapy and/or excision [8, 19–21] with the majority of patients achieving complete remission. Relapse is not infrequent but extracutaneous spread is relatively rare (10%). The outcome is generally excellent with 5-year overall survival quoted at 87% and 5-year disease-specific survival 95% [8, 19–21].

PCFCL is a neoplasm composed of cells that most often resemble centrocytes which are often large. Centroblast-like cells should be in a minority. Tumours composed entirely of centroblasts arranged in diffuse sheets are excluded from this category. PCFCL may show a follicular, follicular and diffuse or purely diffuse pattern of growth [2, 3]. When present, follicles are variably discrete but often ill-defined. Cases with diffuse growth often consist predominately of large centrocytes and can easily be mistaken for diffuse large B cell lymphoma [2, 3]. Spindle cell or sarcomatoid morphology is a well-recognised phenomenon in a small percentage of cases [22, 23]. CD10 positivity is less often found than in nodal follicular lymphomas and is most commonly seen in cases with a follicular architecture, although other antigens associated with germinal centre differentiation, such as stathmin, MEF2B, LMO2, HGAL and BCL6, are almost always expressed [24–27].

Reports on the incidence of BCL2 protein expression and *BCL2* gene rearrangement (*BCL2R*) document disparate results [26, 28, 29]. This is likely to be at least in part related to the sensitivity of the immunohistochemical and molecular techniques employed to detect the protein and rearrangement respectively. Recent studies indicate that weak expression of BCL2 protein is seen in a significant proportion of cases and approximately 10% of cases have *BCL2* rearrangements [24, 30]. False negative immunohistochemical staining for BCL2 protein does not appear to contribute to the low rate of detection, perhaps due to the absence of *BCL2* gene mutations [26]. Staining for CD5, CD23, IRF4/MUM1 and Cyclin D1 is usually negative but rare cases express CD30 [31].

The incidence of *BCL2R* in PCFCL is clearly different from that encountered in nodal follicular lymphoma (nFL). As in nFL, PCFCL also frequently show deletions of 1p36/TNFRSF14 [24, 27]. In PCFCL, loss of 1p36 and *BCL2R* appear to be mutually exclusive, whereas they are often concurrent in nFL [32]. Mutations of *TNFRSF14* are similarly frequent in both entities (30–40%) but the mutational profile of PCFCL differs from that of nFL harbouring *BCL2R* in having lower frequencies of mutations in chromatin modifiers (*CREBBP, KMT2D*, *EZH2*) and *BCL2*, and a higher incidence of mutations in *TNFAIP3* (Table 2) [24]. Nevertheless, the majority of *BCL2R-*PCFCL show high levels of EZH2 expression even in the absence of mutations involving common hotspots [32]. A scoring system to predict secondary involvement in what might appear to be a PCFCL has been proposed. This takes into account mutations in *CREBBP*, *KMT2D*, *EZH2* and *EP300*, *BCL2* rearrangement and a low Ki-67 proliferation index [30]. The mutational profile of PCFCL also shows some overlap with other subtypes of *BCL2R-* FL, particularly those arising at other extranodal sites (Table 2) [24, 33, 34].

**Table 2:**
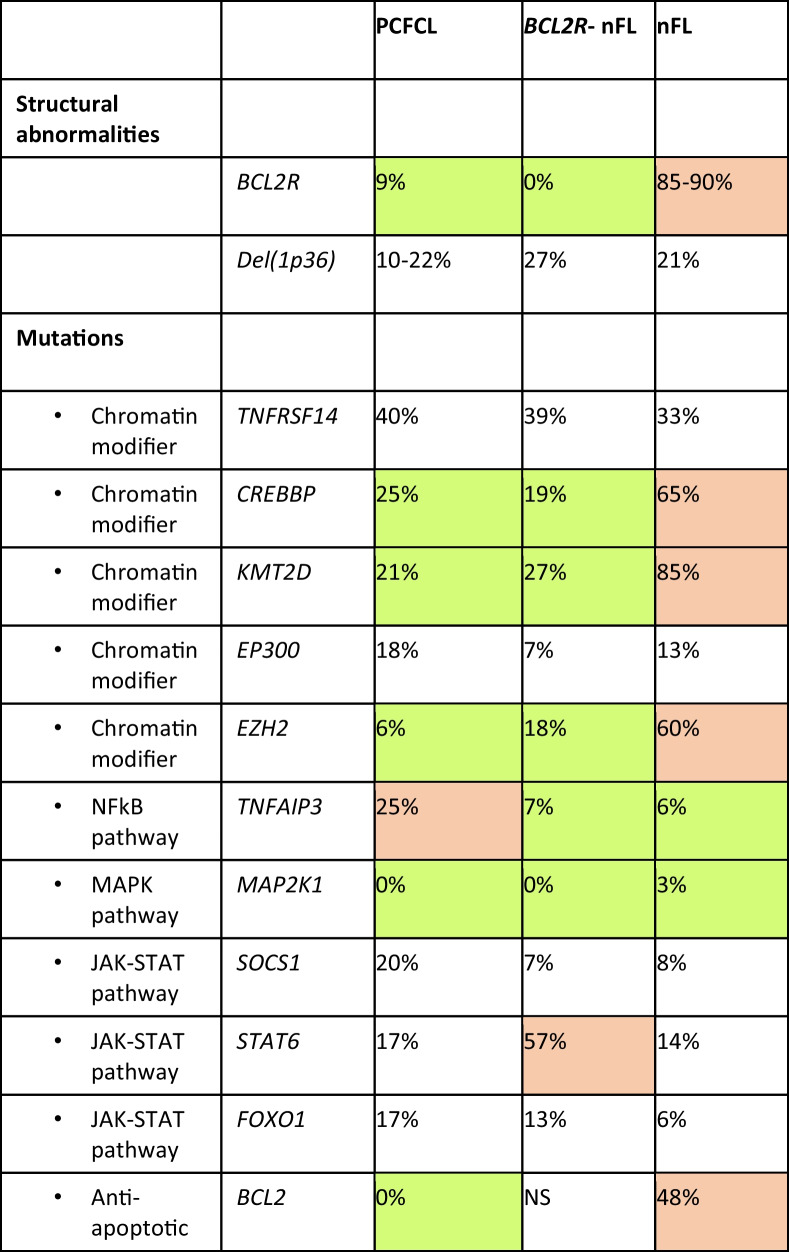
Comparison of structural abnormalities and mutational profiles in different subtypes of follicular lymphoma (incidence figures derived from Barasch et al. [24] and Nann et al. [34])

High incidence relative to other categories of lymphoma highlighted in orange, low incidence relative to other categories highlighted in green

*PCFCL* primary cutaneous follicle centre lymphoma, *BCL2R- nFL* nodal follicular lymphoma lacking BCL2 gene rearrangement *nFL* nodal follicular lymphoma, *NA* not available

Five-year-survivals close to 100% are quoted for PCFCL, even when displaying a diffuse growth pattern and large cell morphology, with only locally directed therapy required to manage the disease [25, 35, 36]. In view of this excellent prognosis, different approach to treatment, frequent lack of *BCL2R* and distinct mutational landscape, PCFCL is retained as a distinct entity, separate from nodal and other types of FL.

### Primary cutaneous diffuse large B cell lymphoma, leg type

Primary cutaneous diffuse large B cell lymphoma, leg type (PCDLBC-LT) was born into controversy but has become established as a distinct entity. It was included in the 1997 EORTC classification, in the EORTC-WHO classification of cutaneous lymphoma in 2005, in subsequent WHO classifications and again in the 2022ICC [2, 3, 5]. PCDLBCL-LT is a tumour of the elderly, most frequently, but not exclusively, presenting on the legs. There is a marked female predominance. Biopsy shows diffuse sheets of centroblast-like and/or immunoblast-like B cells that often fill the dermis. A non-germinal centre phenotype, characterized by an absence of CD10 with positive staining for IRF4/MUM1, strong uniform expression of BCL2 and variable reactivity with antibodies to BCL6 has traditionally been reported. However, in a small minority of cases, CD10 expression and absence of IRF4/MUM1 and/or BCL2 is documented, with a recent study reporting 25% GCB-type cases by Hans algorithm, and 39% by gene expression profiling using paraffin embedded material (with 43% unclassified and 18% activated B cell type) [37, 38]. Thus, the clinical relevance of using these algorithms has limitations and should not be used as an alternative for the current multiparameter approach employed to differentiate PCDLBCL-LT from PCFCL with large cell morphology [38]. Almost all cases of PCDLBCL-LT are IgM positive, in contrast with PCFCL [37, 38]. Rare TdT-positive cases have also been reported, raising a diagnostic dilemma with B lymphoblastic leukaemia/lymphoma which may also present in the skin [39, 40]. Absent or weak expression of CD20 is a clue to the latter.

FISH studies document *MYC* rearrangements in a proportion of cases but *BCL2* translocations should be absent. Cases harbouring both *MYC* and *BCL2* rearrangements are better classified as high-grade B cell lymphoma (HGBCL) with *MYC* and *BCL2* rearrangement. *BCL6* may be rearranged and occasional cases would fulfil the criteria for a HGBCL with *MYC* and *BCL6* rearrangements. Amplification of *BCL2* and deletion of 9p21.3 harbouring *CDKN2A* is common [20, 38, 41].

PCDLBCL-LT has a distinctive mutational profile which overlaps significantly with that of primary CNS, primary testicular DLBCL, intravascular large B cell lymphoma and the MCD group of DLBCL including a high frequency of *MYD88* and *CD79B* mutations (Table 3) [38, 42–44]. In addition, *PIM1* mutations are seen in around 70% of cases and mutation of *MYC* in 20% [37, 42]. Thus, many cases have a molecular/genetic landscape similar to the MCD/C5 group of DLBCL [44].

**Table 3:** Incidence of *MYD88* and *CD79B* mutations in primary cutaneous diffuse large B cell lymphoma, leg type compared to other subtypes of large B cell lymphoma (incidences from Wright et al. [3])

	PCDLBCL-LT	Primary CNS DLBCL	Primary testicular DLBCL	Intravascular LBCL	DLBCL in breast	MCD DLBCL	All nodal DLBCL
*MYD88*	59%	61%	67%	47%	50%	66%	11%
*CD79B*	43%	42%	16%	38%	29%	50%	11%
*MYD88* + *CD79B*	38%	29%	16%	24%	17%	38%	4%

*PCDLBCL*-LT primary cutaneous diffuse large B cell lymphoma, leg type; *DLBCL* diffuse large B cell lymphoma; *LBCL* large B cell lymphoma

During the CAC, there was much debate surrounding the introduction of an umbrella term such as “extranodal lymphoma ABC (non-GCB) type,” at least for some extranodal DLBCL, primarily but not exclusively, including cases arising at sites of immune privilege [5]. However, this was considered premature for the 2022ICC. Whether PCDLBCL-LT should be put under such an umbrella, particularly now with more recent data questioning how many PCDLBCL-LT are of ABC type and with continued strong interest to recognize PCDLBCL-LT as a distinct entity, will remain a question for the future. It is expected that ultimately molecular/cytogenetic classification of all DLBCL may become a standard of practice. One of the main reasons that there is strong support for recognizing PCDLBCL-LT is because it is critical that they be differentiated from PCFCL with predominant large cell morphology and diffuse growth, an entity that might fulfil the diagnosis for a DLBCL, if present at an extracutaneous site. PCFCL have a significantly different prognosis than PCDLBCL-LT and require a radically different therapeutic approach [20, 45].

### Other B cell lymphomas and lymphoproliferative disorders in the skin

EBV-positive mucocutaneous ulcer, discussed in more detail elsewhere in this issue, is a solitary compartmentalized proliferation of EBV infected B cells arising in the context of immune suppression. It may arise primarily in the skin but is most frequently presents in oropharyngeal mucosa.

Although not the focus of this article, a broad range of mature B cell lymphomas may secondarily involve the skin, particularly mantle cell lymphoma, chronic lymphocytic leukaemia/small lymphocytic lymphoma, intravascular large B cell lymphoma (IVLBCL), lymphomatoid granulomatosis, plasmablastic lymphoma and post-transplant lymphoproliferative disorder. Skin limited variants are recognised in some, for example IVLBCL. In addition, B lymphoblastic lymphoma/leukaemia may present primarily in skin.

## Primary cutaneous T cell lymphomas and lymphoproliferative disorders

### Mycosis fungoides and Sézary syndrome

Mycosis fungoides (MF) is the commonest type of cutaneous T cell lymphoma, one that has been recognised as a distinct entity for a very long time but has been segregated from Sézary syndrome (SS) more recently. Clinical presentation is usually with patches and plaques. The clinical course is generally indolent although progression to tumours and/or erythroderma, and even extracutaneous dissemination, may occur in some patients. Historical cases of MF reported to present with tumour nodules from the outset and referred to as tumour d’emblée mycosis fungoides, likely represent other variants of T cell lymphoma, whilst most cases of erythroderma associated with T cell lymphoma display the full clinical picture of SS. Variants of MF recognised as distinct entities are folliculotropic MF and pagetoid reticulosis, together with some others seen less commonly. SS is a rare distinct entity defined by the triad of erythroderma, generalised lymphadenopathy and the presence of clonally related neoplastic T cells in skin, lymph nodes and peripheral blood. SS tends to progress more rapidly and has a worse prognosis than MF [46].

Although classified as separate entities, molecular studies on MF and SS have tended to group these diseases together under the rubric of cutaneous T cell lymphoma. Most studies have focused on SS due to the relative ease with which tumour cells can be accessed in peripheral blood or have focused on more advanced stage MF in view of the relative abundance of tumour cells compared to early stage disease [46]. A full discussion of MF and SS is beyond the scope of this article. However, although detailed knowledge of the initial drivers and promoters of MF and SS is lacking, and despite marked heterogeneity in genomic features of MF and SS, recent studies have identified potentially targetable point mutations in genes that cluster in specific pathways that are worthy of mention. The pathways involved include *JAK-STAT* signalling, *TCR-NFκB* signalling, *MAP* kinase signalling, cell cycle control and apoptosis and chromatin modification. The reader is referred to recent reviews that present such findings in more detail [46, 47].

### Primary cutaneous CD30-positive T cell LPD

Primary cutaneous CD30-positive T ell lymphoproliferative disorders encompass a spectrum of disease with overlapping histological, immunophenotypic and genetic features, lymphomatoid papulosis (LyP) at one end and primary cutaneous anaplastic large cell lymphoma (pcALCL) at the other. Collectively, LyP and pcALCL are the second most commonly encountered subtype of primary cutaneous lymphoma/lymphoproliferative disorder, accounting for approximately 30% cases [7, 48].

LyP classically presents as crops of papules and nodules which develop, become haemorrhagic and ulcerate over 3 to 4 weeks, then spontaneously resolve. Resolution may take up to 12 weeks and leave varioliform scars. In children, lesions may present as rapidly enlarging ulcerated nodules, usually on a background of more typical lesions [49]. In a small subset of patients with angioinvasive growth (see below), papules develop and ulcerate, forming large eschar-like ulcers that may measure several centimetres in diameter. Although these spontaneously resolve, there is prominent residual scarring [50]. Approximately 10–20% of patients with LyP also develop MF, either concurrently, prior to, or following the diagnosis of LyP [49]. LyP and pcALCL may also co-exist in the same patient [51]. In contrast to LyP, the patients with pcALCL present with nodules or tumours that are larger than those seen in LyP and do not show waxing and waning, although spontaneous resolution is seen in a minority of cases [52]. To qualify as pcALCL, > 75% of tumour cells should be CD30 positive [2, 53]. In the majority of cases (80%), the lesions are solitary or localised but multifocal disease may be encountered. Cases which microscopically display pseudoepitheliomatous hyperplasia may clinically mimic squamoproliferative lesions such as keratoacanthoma or squamous cell carcinoma. LyP and pcALCL are indolent diseases apart from exceedingly rare cases of the latter which exhibit early widespread dissemination [54]. Leg involvement is also reported to be an adverse prognostic indicator and requires special attention [52].

Since first described, the morphological spectrum of LyP has been considerably expanded. Various histological patterns or types of LyP have been described and designated LyP types A, B, C, D, E and *DUSP22* rearranged [7, 48]. Type A LyP displays prototypic morphology and is composed of large CD30-positive T cells with anaplastic features on a background of mixed inflammatory cells. Type C LyP is similar but contains more numerous CD30-positive cells, often forming cohesive sheets and morphologically mimicking pcALCL with clinical information required to make the distinction. LyP types B and D are epidermotropic; type B cases are CD4 positive and mimic MF whilst the tumour cells in type D express CD8. Angiocentric and angioinvasive growth is the hallmark of type E LyP. By definition, lesional cells express CD30, apart from some cases of type B LyP which have been reported to lack this antigen. Most cases express CD4 and cytotoxic molecules, although variations are seen. These include CD8 expression, which often correlates with variant morphology in LyP (i.e. types D and E as well as *DUSP22-R* LyP)[48].

The sixth type of LyP is defined by the presence of *DUSP22* rearrangement and characterized by a biphasic morphology with a prominent epidermotropic component of small to medium sized lymphocytes that weakly express CD30, overlying nodular aggregates of large lymphoid cells that are strongly positive for CD30 [55]. Rearrangement of the *DUSP22* gene is also seen in a subset of systemic ALK-negative anaplastic large cell lymphoma (ALK-ALCL) and in about 20% of pcALCL [55–57]. Many but not all of the latter cases have similar pathological features to those with *DUSP22* rearranged LYP [55, 58]. The 2022ICC recommends recognition of *DUSP22* rearranged (*DUSP22-R*) systemic ALK-ALCL as a subtype of ALK-ALCL in view of distinct morphologic, phenotypic, genomic and epigenetic features [5]. However, the clinical behaviour of pcALCL with and without *DUSP22-R* is similar, precluding a necessity to test for it routinely in clinical practice. Whilst some studies suggest that, in MF patients, the presence of *DUSP22-R* might help differentiate concurrent primary cutaneous CD30-positive LPD from large cell transformation, others cast doubt on the utility of this, *DUSP22-R* being found in two of eleven cases of large cell transformation of mycosis fungoides (LCT-MF) in one study [57, 59]. *TP63* rearrangements, associated with poor prognosis in systemic ALK-ALCL, have also rarely been found in pcALCL but the paucity of such cases studied to date preclude comment on the clinical significance of this finding [60].

Rearrangement of the *ALK* gene has traditionally been equated with systemic ALK-positive ALCL, even when presenting in the skin. However, a small number of bona fide cases of ALK-positive ALCL confined to the skin have been reported, predominantly in paediatric patients [61, 62]. Whether this represents early stage ALK^+^ ALCL presenting in skin or a distinct type of cutaneous ALCL has yet to be determined. Lastly, in a study of 47 primary cutaneous CD30-positive LPDs, *NPM1-TYK2* gene fusion was been identified in one case each of LyP and pcALCL [63].

As in MF and SS, mutations affecting the *JAK1-STAT3* signalling pathway are identified in primary cutaneous CD30-positive LPDs but only in a small minority of cases (5%) [47, 64]. Epigenetic alterations are also noted, including upregulation of *SATB1* in nearly all cases of LyP and in approximately a third of pcALCL as well as differential expression of several miRNAs compared to normal and systemic ALCL [47, 65, 66].

LyP and pcALCL frequently co-exist in the same patient and are clonally related [67, 68]. Further, 10–20% of LyP patients also develop MF and, in most cases, both processes are part of the same neoplastic clone [49, 51]. A recent report performed array-based comparative genomic hybridization and next-generation sequencing on clonally related lesions of LyP, pcALCL and a nodal deposit of ALCL in the same patient. These demonstrated a low burden of abnormalities in LyP compared to pcALCL, implying relative genetic stability in the former. Moreover, different genomic abnormalities were present in different lesions despite belonging to the same clone, suggesting early divergence from a common precursor [69].

### Primary cutaneous CD4-positive small/medium T cell lymphoproliferative disorder

**Fig. 3:**
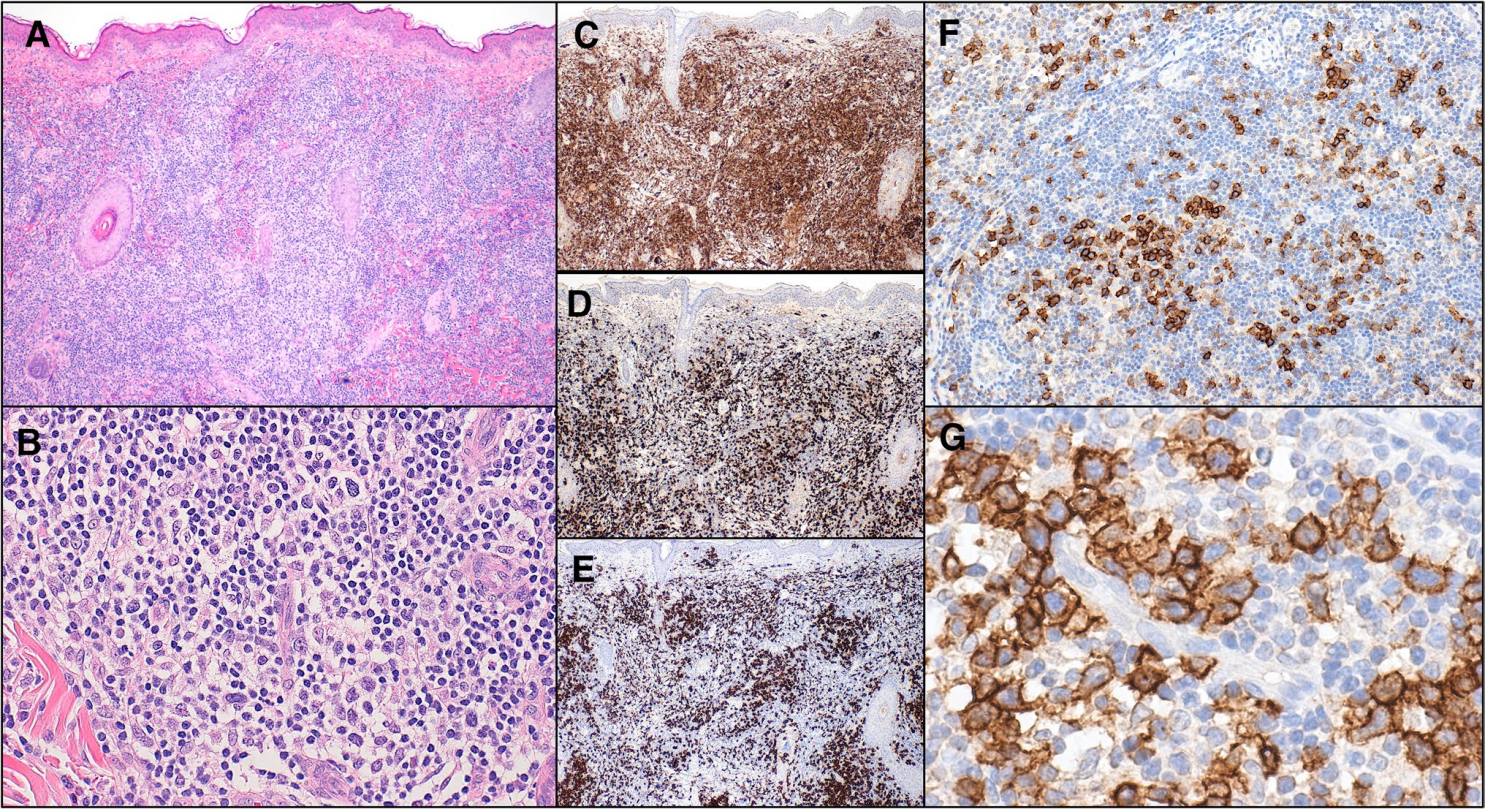
Primary cutaneous CD4^+^ small/medium T cell lymphoproliferative disorder. **A** Diffuse dermal infiltrate seen at low magnification. **B** Ill-defined pale-staining aggregates of cells with intermediate to large irregular nuclei seen at higher magnification. **C** CD4^+^ T cells predominate. **D** Many CD8^+^ T cells are also present. **E** Numerous CD20^+^ B cells are also seen. **F** Loose aggregates of bright CD279/PD1^+^ cells scattered throughout. **G** High magnification view of CD279/PD1^+^ cells show that they correspond to the larger cells with irregular nuclear contours

The term primary cutaneous CD4-positive small/medium T cell lymphoproliferative disorder (CD4^+^ SMTLPD) was a provisional entity in the in the 2017 revision of the 4th edition of the WHO classification of haematological malignancy. It is somewhat poorly understood in some quarters and not well defined, raising the possibility of misdiagnosis, unnecessary staging investigations and over-treatment. Although at odds with the terminology associated with this entity, the defining feature is the presence of small clusters of brightly CD279/PD1-positive CD4-positive T cells that are mostly of intermediate to large size but with less than 30% large lymphoid cells. They possess irregular nuclei and form small clusters scattered against a background of reactive small CD4-positve and CD8-positive T cells, B lymphocytes, histiocytes and variable numbers of plasma cells, neutrophils and eosinophils (Fig. 3) [70–74]. Clonality is found in the majority of cases but little else is known regarding the molecular landscape of this disorder.

Use of this term should be limited to solitary or localized lesions confined to the skin in the absence of patches and plaques. This scenario correlates with a benign clinical course with 5-year survivals of 100% quoted [70–75]. Consequently, the entity is termed a lymphoproliferative disorder rather than a lymphoma. A more cautious approach should be adopted when lesions are large, rapidly growing and/or multiple, when there is a significantly aberrant phenotype with loss of CD2, CD3 or CD5, and when few reactive CD8-positive T cells or B cells are present in the infiltrate [71].

### Primary cutaneous CD8-positive aggressive epidermotropic cytotoxic T cell lymphoma

Primary cutaneous CD8-positive aggressive epidermotropic cytotoxic T cell lymphoma (CD8^+^ AECTL) is a rare but aggressive disease of adults. Clinical-pathological correlation is essential in the diagnosis of this entity. It shows significant morphological overlap with other CD8-positive epidermotropic lymphomas and is largely distinguished on the basis of distinctive clinical features, again emphasizing the critical role of clinical correlation in defining the cutaneous lymphomas (Fig. 5) [76–78]. Patients present with a generalised eruption of rapid onset, with development of multiple indurated plaques and crusted ulcerated tumours without a preceding history of slowly evolving patches. Early dissemination to other extranodal sites, such as testis, lungs, spleen and CNS, often occurs although lymph node involvement is uncommon. The prognosis is dismal and there is no standard treatment, although usually CHOP-like regimens are administered. A median survival of 12 months is reported [79].

As the name suggests, lesions are characterized by a prominent epidermotropic infiltrate of CD8-positive T cells that are usually of medium to large size with large irregular hyperchromatic or blast-like nuclei. Epidermal necrosis is frequent, a dermal infiltrate is usually present and angiocentricity and angioinvasion are common [70–74]. The neoplastic T cells are of alpha–beta type (TCR-βF1 positive) and express cytotoxic molecules in addition to CD8. Unlike many other cutaneous T cell lymphoma, CD7 is usually positive along with CD3. CD2 and CD5 are frequently lost. Staining for CD30 and EBV is also negative.

Currently, there are no molecular markers that facilitate diagnosis of this disease. However, recent high-resolution genomic analysis has shed some light on the pathogenesis of CD8^+^ AECTL [80]. In particular, overactivity of *JAK2* signalling appears to play a central role. CD8^+^ AECTL have a heterogeneous and complex genomic landscape, and share many pathogenetic features with MF and SS. However, unlike other cutaneous T cell lymphomas, they harbour rearrangements of *JAK2*, predicted to induce constitutive upregulation of kinase activity, or inactivating deletions or rearrangements of *SH2B3*, a negative regulator of the JAK-STAT pathway. *JAK2* and *SH2B3* abnormalities are mutually exclusive and found in 75% of patients with CD8^+^ AECTL [80]. As well as confirming upregulation of *JAK-STAT* signalling, gene expression analysis of these cases also showed that co-activation of the *NFκB* pathway, along with upregulated *JAK-STAT* signalling, is likely to induce a pro-oncogenic inflammatory microenvironment in CD8^+^ AETCL.

### Primary cutaneous acral CD8+ T cell lymphoproliferative disorder

Primary cutaneous acral CD8^+^ T cell lymphoproliferative disorder (CD8^+^ TLPD) has only recently been recognised. First described as an indolent CD8-positive lymphoid proliferation on the ear, the clinical spectrum has subsequently been expanded to reflect its distribution at other acral sites [81–83]. Although initial reports referred to this as a lymphoproliferative disorder, the nomenclature was change to primary cutaneous acral CD8-positive T cell lymphoma in the 2017 revision of the 4th edition of the WHO classification [84]. Whilst retaining designation as a CD8-positive lesion of acral sites, the 2022ICC preferred to revert to calling this entity a lymphoproliferative disorder in view of its indolent behaviour, and in keeping with a trend to more conservative use of nomenclature across categories. It is now also recognised as a definite rather than provisional entity [5].

**Fig. 4:**
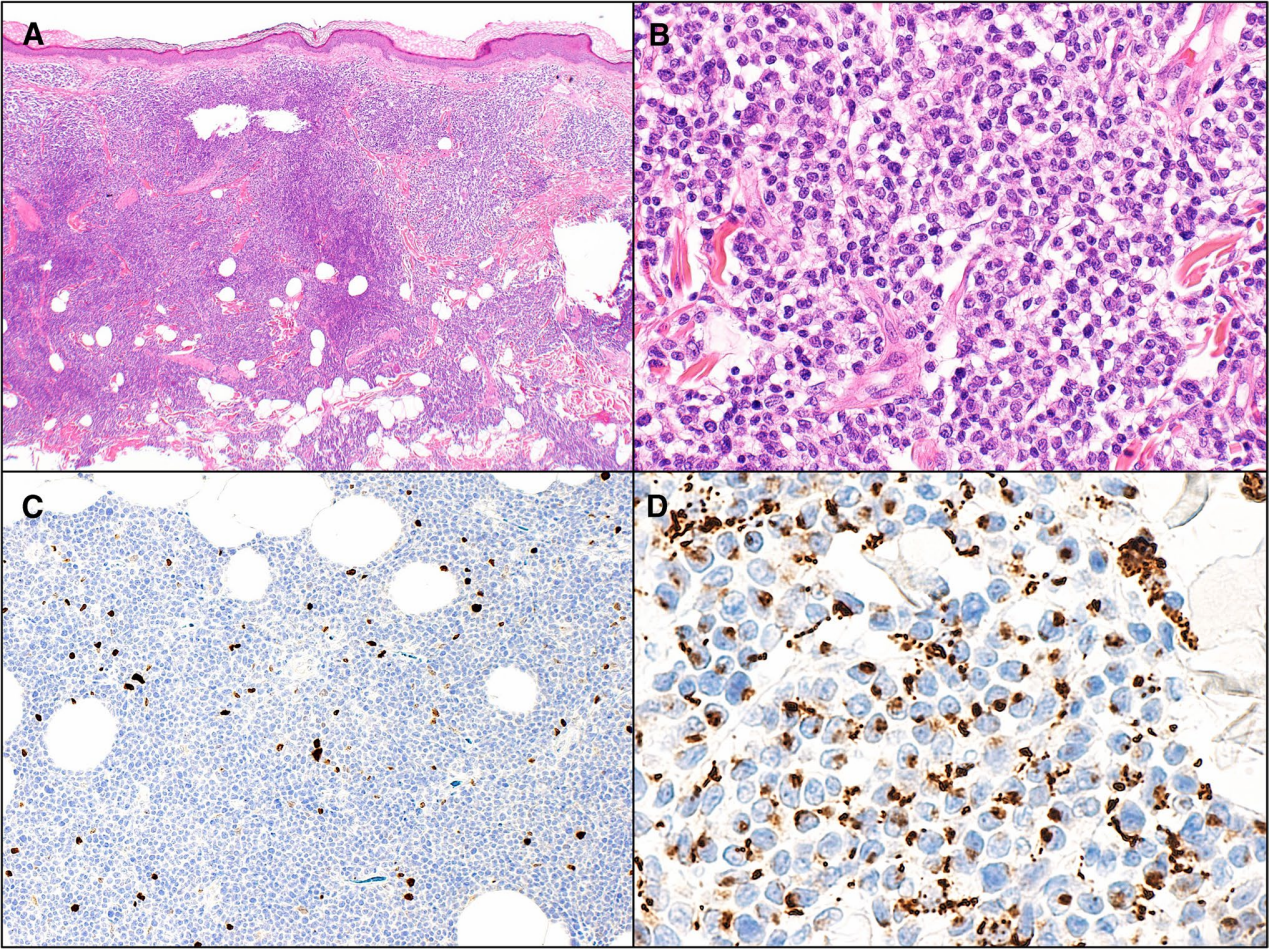
Primary cutaneous acral CD8^+^ lymphoproliferative disorder. **A** Low magnification view showing a diffuse infiltrate filling the dermis and extending into subcutis. **B** Higher magnification view demonstrating monotonous appearance of tumour cells. **C** Ki67 staining showing typical low proliferation index. **D** Perinuclear Golgi-dot staining with antibodies to CD68 is a useful diagnostic clue

CD8^+^ TLPD presents as a slow growing, reddish/purple nodule or plaque in adults that can measure up to several centimetres in maximal dimension and most frequently present in the facial region, often on the ear or nose, but also on the hands and feet. Lesions are almost always solitary although multiple lesions, including bilateral on the ears and feet, have been reported [81, 82, 85–87]. Biopsy shows a diffuse, non-epidermotropic dermal infiltrate that typically spares adnexal structures and may involve subcutaneous fat [82, 86]. The infiltrate is composed of a uniform population of intermediate sized lymphoid cells with irregular nuclei, finely dispersed chromatin and small to medium nucleoli (Fig. 4). Mitotic figures are sparse and there is no angioinvasion or necrosis. By definition, the neoplastic lymphoid cells are TCR-βF1 positive T cells that express CD8 and TIA-1. They are usually CD3 positive but often lack one or more of CD2, CD5 and CD7. Staining for CD30, CD56, CD57, TdT and EBV are negative. Antibodies to CD68 show an unusual Golgi-dot pattern of staining which is not seen generally seen in other types of cutaneous T cell lymphoma [86, 87]. This, together with a uniformly low Ki67 index, is a useful diagnostic clue (Fig. 4). Cases are clonal but little else is know about the molecular landscape of this enigmatic tumour.

**Fig. 5:**
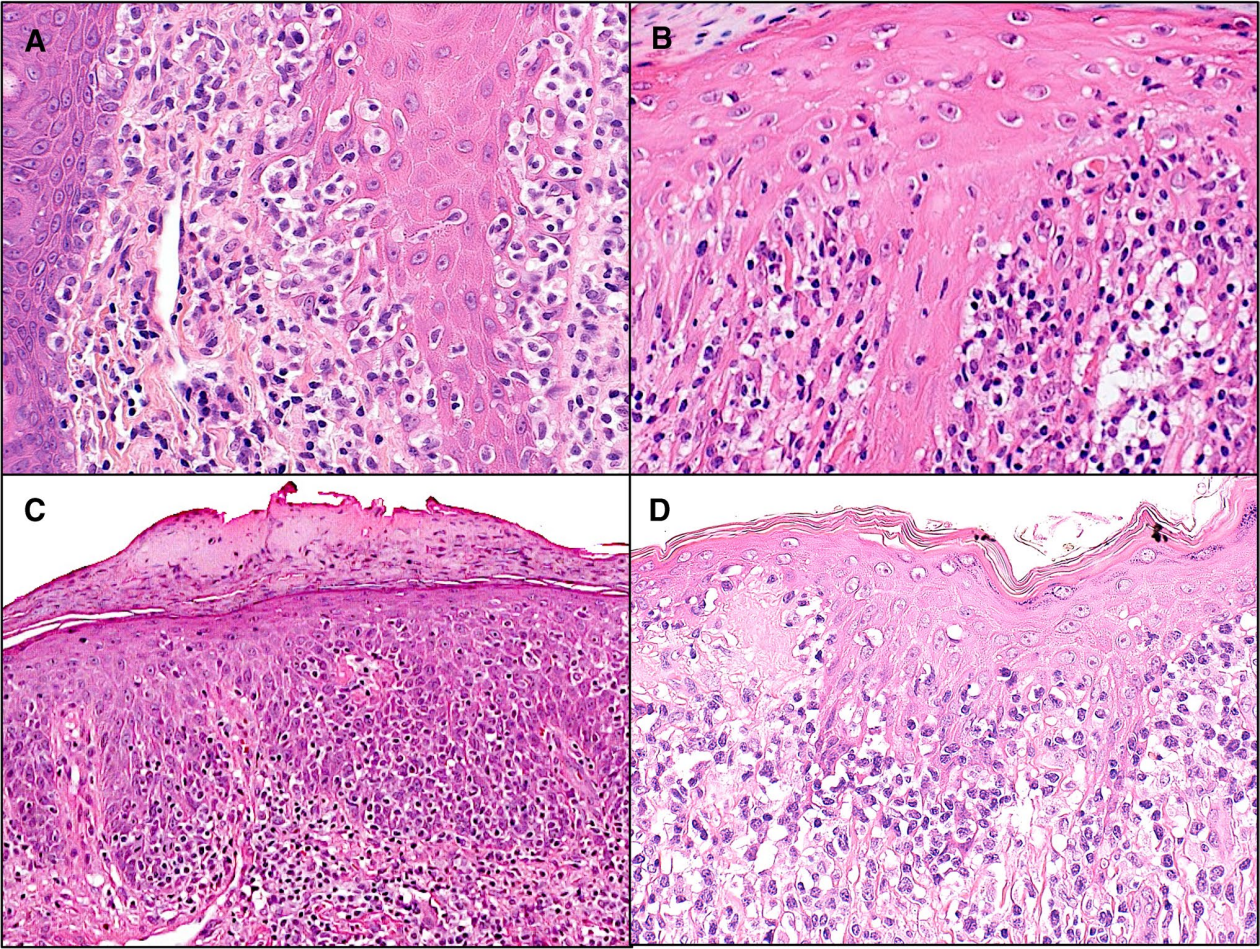
Epidermotropism is not exclusive to mycosis fungoides but can be seen in a variety of cutaneous T cell lymphomas. Examples include **A** Pagetoid reticulosis, **B** Lymphomatoid papulosis type-D, **C** primary cutaneous CD8-positive aggressive epidermotropic cytotoxic T cell lymphoma, and **D** primary cutaneous T cell lymphoma. As well as shared morphology, there is often significant phenotypic overlap amongst these entities. Clinical correlation is therefore essential for accurate diagnosis and ensuring appropriate treatment

Despite clonality and an aberrant phenotype, the prognosis is excellent. There may be cutaneous relapses but extracutaneous dissemination rarely, if ever occurs. Even though most reported cases have been managed with non-aggressive therapy, there have been no disease related deaths to date [86]. Surgical excision and/or local radiotherapy appear to be effective treatments (Fig. 5).

### Subcutaneous panniculitis-like T cell lymphoma

The infiltrate in SCPTL does not involve the dermis or epidermis, being restricted to fat lobules with relative sparing of the septae. The neoplastic lymphocytes characteristically rim adipocytes with their nuclei protruding into the fat space (a “lacelike” pattern). They possess irregular hyperchromatic nuclei and pale cytoplasm, and are associated with prominent nuclear karryorhexis, fat necrosis and numerous histiocytes (cytophagia or “bean-bag-like” cells). Other inflammatory cells, particularly plasma cells, are absent, although angioinvasion has been described [88, 90]. The tumour cells are TCR-βF1 positive T cells that express CD8, TIA-1, granzyme B and perforin. There is variable loss of CD2, CD5 and CD7 and staining for CD30, CD56 and EBV is negative. Antibodies to Ki67 are useful in highlighting neoplastic lymphocytes rimming individual adipocytes. This is not a feature of SLE, nor would be a paucity of plasma cells.

Germline mutations of the *HAVCR2* gene have recently been documented in a high percentage of patients with SCPTL [92–94]. *HAVCR2* codes for T cell immunoglobulin and mucin domain-containing protein 3 (TIM3). TIM3 is an inhibitory receptor expressed on interferon-γ producing T-cells and plays a key role in regulating inflammation [95]. Mutations of *HAVCR2* result in misfolding of TIM3, abrogating plasma membrane expression [95]. *HAVCR2*^Y82C^ (c.245A > G, p.Tyr82Cys) is found in ~ 85% of patients with SCPTL of East Asian or Polynesian ancestry whilst *HAVCR2*^I97M^ (c.291A > G, p.Lle97Met) is present in ~ 25% of patients of European descent [92–94]. Compared to those with wild-type *HAVCR2*, patients with *HAVCR2*^*MU*T^ SCPTCL tend to be younger (< 30 years), more often have associated hemophagocytic lymphohistiocytosis and have a shorter relapse-free survival. *HVACR2*^*MUT*^ SCPTL have upregulation of genes associated with the TNFα-NFκB pathway, IL6-JAK-STAT3 pathway, MTOR signalling pathway and apoptosis, whilst genes associated with lymphocyte homing (*CCR4*, *GPR183*) and autoimmunity are upregulated in *HVACR2*^*WT*^ SCPTCL [93]. These results not only enhance our understanding of the pathogenesis of SCPTL but may also allow for risk stratification to aid therapeutic decision making and may ultimately provide a target for novel therapies.

### Primary cutaneous gamma-delta T cell lymphoma

Primary cutaneous T cell lymphoma (PCGDTL) were grouped along with SCPTL in early classifications of skin lymphoma but have subsequently been segregated, recognised as a provisional entity in the 4th edition of the WHO classification and a definite entity in the revised 4th edition and the 2022ICC [2, 84, 90]. PCGDTL is a very rare tumour mostly affecting adults [84, 90]. Lesions are widespread at presentation and include patches, plaques, nodules and tumours, the latter often ulcerated [90, 96, 97]. Lymph nodes, spleen and bone marrow involvement is unusual but there is frequent dissemination to mucosal and other extranodal sites [90, 96, 97]. B-symptoms are common and approximately 25% of patients develop haemophagocytic lymphohistiocytosis [90, 96, 97]. Multiagent chemotherapy and radiotherapy are the treatment of choice although response to treatment is often poor: median survival is 31 months and the 5-year survival 19.9% [90, 96, 97].

Tumour cells are usually medium to large in size with coarsely clumped chromatin [98]. Patterns of cutaneous infiltration are varied with epidermotropic, dermal and subcutaneous variants, often correlating with the clinical appearance: epidermotropism often correlates with patches and plaques whilst dermal and subcutaneous infiltrates more often correlate with tumours. Angioinvasion and necrosis may be encountered. By definition, the tumour cells have a phenotype and this should be confirmed using antibodies to TCR-γ or TCR-δ. Staining for TCR-βF1 is negative. CD5 is characteristically absent but other T -cell associated antigens such as CD2, CD3 and CD7 retained. Most cases are negative for CD4 and CD8 but show strong expression of cytotoxic molecules. CD56 is frequently but not always positive, particularly in subcutaneous infiltrates. Staining for EBV is negative [90–98].

TCR-γ or TCR-δ genes are clonally rearranged. The TCR-β gene may also be clonally rearranged but is never expressed [96]. γ/δT cells normally express one of three separate Vδ isoforms as part of the T cell receptor heterodimer. Vδ1 γ/δT cells are predominantly found in mucosal sites whilst Vδ2 γ/δT cells constitute the majority of circulating γ/δT cells. A recent study has shown that epidermotropic and/or dermal centred PCGDTCL derive from Vδ1 γ/δT cells, whereas those with a panniculitis-like pattern of infiltration are of Vδ2 γ/δT cell origin. In addition, TCR chain usage appears to be non-random. All the Vδ2 lymphomas studied were accompanied by a Vγ3 chain, an uncommon finding in the peripheral blood whilst the vast majority of Vδ1 lymphomas had an accompanying Vγ3 or Vγ5 chain. On this basis, the authors postulate a common antigenic trigger for each tumour subtype [99]. Irrespective of cell of origin, all PCGDTL displayed similar genetic landscapes with potentially targetable mutations in the *JAK/STAT*, *MAPK*, *MYC*, and chromatin modification pathways.

### Other T and NK-cell lymphomas and lymphoproliferative disorders in the skin

Hydroa vacciniforme lymphoproliferative disorder in an EBV-positive proliferation of T or NK-cells that affects the skin and is seen predominantly in children and adolescents from Asia and Latin America. It is included amongst the group of virally related mature T and NK-cell neoplasms along with severe mosquito bite allergy, chronic active EBV disease, and systemic EBV-positive T cell lymphoma of childhood, and is discussed elsewhere within this issue. Other T and NK-cell lymphomas with a propensity to infiltrate skin include extranodal NK/T-cell lymphoma, nasal type, the T/ NK-cell variant of intravascular large cell lymphoma, angioimmunoblastic T cell lymphoma and adult T cell leukaemia/lymphoma. A detailed discussion of these entities is beyond the scope of this article.

Peripheral T cell lymphoma, NOS is applied to cutaneous lymphomas not fulfilling diagnostic criteria of the well-defined entities described above, even in the absence of extracutaneous disease. Some of these are CD4 positive and may express markers of T follicular helper cell differentiation, causing diagnostic confusion with CD4^+^ SMTLPD. However, patients typically present with multiple lesions and show a tendency to progress [100, 101]. It remains to be resolved how to best classify this heterogeneous group of neoplasms.

## Summary/conclusions

In the 2022ICC, the terminology used has changed for some cutaneous lymphoid neoplasms and the diagnostic criteria been refined for others. In addition, the increasing application of advanced technology to meticulously investigate well-defined and clinically annotated cohorts of cases is beginning to shed light on the pathogenetic mechanisms at play in many of these diseases and will continue to do so. The results of such studies reinforce already established differences between some of the entities currently recognised but also raise questions about how we categorise others, particularly with respect to relationships which may or may not exist with similar lymphomas at extracutaneous sites. Moreover, a better understanding of the genetic, epigenetic and transcriptional changes in this group of diseases is beginning to identify potential targets for novel therapies, some of which may be relevant across subtypes. Targeting of the *JAK-STAT* signalling pathway is of potential therapeutic value in a range of cutaneous T cell lymphomas, including MF/SS, primary cutaneous CD30-positive LPD, CD8^+^ AECTL, SCPTL and PCGDTCL. Other considerations include *NFκB* signalling in MF/SS, SCPTL and PCGDTL, chromatin modification in MF/SS and PCGDTL and B cell receptor signalling in PCDLBCL-LT. Candidate drugs are already licensed for other haematological malignancies and their use in the field of cutaneous lymphoma merits further exploration, although construction of appropriately powered clinical studies may be a rate limiting factor. How we treat specific diseases and how we predict response to specific therapies will also likely increasingly factor in to how we classify them. Thus, whilst there have been relatively few changes in current classification of primary cutaneous lymphomas, the stage is set for a more rapid evolution in taxonomy in the near future.

## References

[1] Jaffe ESHN, Stein H, Vardiman JW (2001). World Health Organization Classification of Tumours: Pathology and Genetics of Tumours of Haematopoietic and Lymphoid Tissues.

[2] Willemze R, Kerl H, Sterry W, Berti E, Cerroni L, Chimenti S, Diaz-Perez JL, Geerts ML, Goos M, Knobler R, Ralfkiaer E, Santucci M, Smith N, Wechsler J, van Vloten WA, Meijer CJ (1997). EORTC classification for primary cutaneous lymphomas: a proposal from the Cutaneous Lymphoma Study Group of the European Organization for Research and Treatment of Cancer. Blood.

[3] Willemze R, Jaffe ES, Burg G, Cerroni L, Berti E, Swerdlow SH, Ralfkiaer E, Chimenti S, Diaz-Perez JL, Duncan LM, Grange F, Harris NL, Kempf W, Kerl H, Kurrer M, Knobler R, Pimpinelli N, Sander C, Santucci M, Sterry W, Vermeer MH, Wechsler J, Whittaker S, Meijer CJ (2005). WHO-EORTC classification for cutaneous lymphomas. Blood.

[4] Arber DA, Orazi A, Hasserjian RP, Borowitz MJ, Calvo KR, Kvasnicka HM, Wang SA, Bagg A, Barbui T, Branford S, Bueso-Ramos CE, Cortes J, Dal Cin P, DiNardo CD, Dombret H, Duncavage EJ, Ebert BL, Estey E, Facchetti F, Foucar K, Gangat N, Gianelli U, Godley LA, Goekbuget N, Gotlib JR, Hellström-Lindberg E, Hobbs G, Hoffman R, Jabbour EJ, Kiladjian JJ, Larson RA, Le Beau MM, Loh ML, Löwenberg B, Macintyre EA, Malcovati L, Mullighan CG, Niemeyer CM, Odenike O, Ogawa S, Orfao A, Papaemmanuil E, Passamonti F, Porkka K, Pui CH, Radich JP, Reiter A, Rozman M, Rudelius M, Savona MR, Schiffer C, Schmitt-Graeff A, Shimamura A, Sierra J, Stock W, Stone RM, Tallman MS, Thiele J, Tien HF, Tzankov A, Vannucchi AM, Vyas P, Wei AH, Weinberg OK, Wierzbowska A, Cazzola M, Döhner H, Tefferi A (2022). International Consensus Classification of Myeloid Neoplasms and Acute Leukemia: integrating morphological, clinical, and genomic data. Blood.

[5] Campo E, Jaffe ES, Cook JR, Quintanilla-Martinez L, Swerdlow SH, Anderson KC, Brousset P, Cerroni L, de Leval L, Dirnhofer S, Dogan A, Feldman A, Fend F, Friedberg JW, Gaulard P, Ghia P, Horwitz SM, King RL, Salles GA, San-Miguel JF, Seymour JF, Treon SP, Vose J, Zucca E, Advani R, Ansell SM, Au WY, Barrionuevo C, Bergsagel PL, Chan WC, Cohen JI, d'Amore F, Davies AJ, Falini B, Ghobrial IM, Goodlad JR, Gribben JG, Hsi ED, Kahl BS, Kim WS, Kumar SK, LaCasce AS, Laurent C, Lenz G, Leonard JP, Link MP, López-Guillermo A, Mateos MV, Macintyre EA, Melnick AM, Morschhauser F, Nakamura S, Narbaitz M, Pavlovsky A, Pileri SA, Piris MA, Pro B, Rajkumar SVV, Rosen ST, Sander B, Sehn LH, Shipp MA, Smith SM, Staudt LM, Thieblemont C, Tousseyn T, Wilson WH, Yoshino T, Zinzani PL, Dreyling M, Scott DW, Winter JN, Zelenetz AD (2022). The International Consensus Classification of Mature Lymphoid Neoplasms: a report from the Clinical Advisory Committee. Blood.

[6] Leboit P.E. BG, Weedon D., Sarasin A (eds) (2006) Pathology and Genetics of Skin Tumours. IARC, Lyon

[7] Willemze R, Cerroni L, Kempf W, Berti E, Facchetti F, Swerdlow SH, Jaffe ES (2019). The 2018 update of the WHO-EORTC classification for primary cutaneous lymphomas. Blood.

[8] Senff NJ, Hoefnagel JJ, Jansen PM, Vermeer MH, van Baarlen J, Blokx WA, Canninga-van Dijk MR, Geerts ML, Hebeda KM, Kluin PM, Lam KH, Meijer CJ, Willemze R (2007). Reclassification of 300 primary cutaneous B-Cell lymphomas according to the new WHO-EORTC classification for cutaneous lymphomas: comparison with previous classifications and identification of prognostic markers. J Clin Oncol : Off J Am Soc Clin Oncol.

[9] Servitje O, Muniesa C, Benavente Y, Monsálvez V, Garcia-Muret MP, Gallardo F, Domingo-Domenech E, Lucas A, Climent F, Rodriguez-Peralto JL, Ortiz-Romero PL, Sandoval J, Pujol RM, Estrach MT (2013). Primary cutaneous marginal zone B-cell lymphoma: response to treatment and disease-free survival in a series of 137 patients. J Am Acad Dermatol.

[10] Gibson SE, Swerdlow SH (2020). How i diagnose primary cutaneous marginal zone lymphoma. Am J Clin Pathol.

[11] Brenner I, Roth S, Puppe B, Wobser M, Rosenwald A, Geissinger E (2013). Primary cutaneous marginal zone lymphomas with plasmacytic differentiation show frequent IgG4 expression. Mod Pathol.

[12] Carlsen ED, Swerdlow SH, Cook JR, Gibson SE (2019). Class-switched primary cutaneous marginal zone lymphomas are frequently IgG4-positive and have features distinct from IgM-positive cases. Am J Surg Pathol.

[13] Carlsen ED, Bhavsar S, Cook JR, Swerdlow SH (2022). IRTA1 positivity helps identify a MALT-lymphoma-like subset of primary cutaneous marginal zone lymphomas, largely but not exclusively defined by IgM expression. J Cutan Pathol.

[14] Edinger JT, Kant JA, Swerdlow SH (2010). Cutaneous marginal zone lymphomas have distinctive features and include 2 subsets. Am J Surg Pathol.

[15] van Maldegem F, van Dijk R, Wormhoudt TA, Kluin PM, Willemze R, Cerroni L, van Noesel CJ, Bende RJ (2008). The majority of cutaneous marginal zone B-cell lymphomas expresses class-switched immunoglobulins and develops in a T-helper type 2 inflammatory environment. Blood.

[16] Schreuder MI, Hoefnagel JJ, Jansen PM, van Krieken JH, Willemze R, Hebeda KM (2005). FISH analysis of MALT lymphoma-specific translocations and aneuploidy in primary cutaneous marginal zone lymphoma. J Pathol.

[17] Streubel B, Simonitsch-Klupp I, Mullauer L, Lamprecht A, Huber D, Siebert R, Stolte M, Trautinger F, Lukas J, Puspok A, Formanek M, Assanasen T, Muller-Hermelink HK, Cerroni L, Raderer M, Chott A (2004). Variable frequencies of MALT lymphoma-associated genetic aberrations in MALT lymphomas of different sites. Leukemia.

[18] Maurus K, Appenzeller S, Roth S, Kuper J, Rost S, Meierjohann S, Arampatzi P, Goebeler M, Rosenwald A, Geissinger E, Wobser M (2018). Panel sequencing shows recurrent genetic FAS alterations in primary cutaneous marginal zone lymphoma. J Invest Dermatol.

[19] Hoefnagel JJ, Vermeer MH, Jansen PM, Heule F, van Voorst Vader PC, Sanders CJ, Gerritsen MJ, Geerts ML, Meijer CJ, Noordijk EM, Willemze R (2005). Primary cutaneous marginal zone B-cell lymphoma: clinical and therapeutic features in 50 cases. Arch Dermatol.

[20] Senff NJ, Noordijk EM, Kim YH, Bagot M, Berti E, Cerroni L, Dummer R, Duvic M, Hoppe RT, Pimpinelli N, Rosen ST, Vermeer MH, Whittaker S, Willemze R (2008). European Organization for Research and Treatment of Cancer and International Society for Cutaneous Lymphoma consensus recommendations for the management of cutaneous B-cell lymphomas. Blood.

[21] Willemze R, Hodak E, Zinzani PL, Specht L, Ladetto M (2013). Primary cutaneous lymphomas: ESMO Clinical Practice Guidelines for diagnosis, treatment and follow-up. Annals Oncol : Off J Eur Soc Med Oncol.

[22] Charli-Joseph Y, Cerroni L, LeBoit PE (2015). Cutaneous spindle-cell B-cell lymphomas: most are neoplasms of follicular center cell origin. Am J Surg Pathol.

[23] Goodlad JR (2001). Spindle-cell B-cell lymphoma presenting in the skin. Br J Dermatol.

[24] Barasch NJK, Liu YC, Ho J, Bailey N, Aggarwal N, Cook JR, Swerdlow SH (2020). The molecular landscape and other distinctive features of primary cutaneous follicle center lymphoma. Hum Pathol.

[25] Goodlad JR, Krajewski AS, Batstone PJ, McKay P, White JM, Benton EC, Kavanagh GM, Lucraft HH (2002). Primary cutaneous follicular lymphoma: a clinicopathologic and molecular study of 16 cases in support of a distinct entity. Am J Surg Pathol.

[26] Szablewski V, Ingen-Housz-Oro S, Baia M, Delfau-Larue MH, Copie-Bergman C, Ortonne N (2016). Primary cutaneous follicle center lymphomas expressing BCL2 protein frequently harbor BCL2 gene break and may present 1p36 deletion: a study of 20 cases. Am J Surg Pathol.

[27] Verdanet E, Dereure O, René C, Tempier A, Benammar-Hafidi A, Gallo M, Frouin E, Durand L, Gazagne I, Costes-Martineau V, Cacheux V, Szablewski V (2017). Diagnostic value of STMN1, LMO2, HGAL, AID expression and 1p36 chromosomal abnormalities in primary cutaneous B cell lymphomas. Histopathology.

[28] Cerroni L, Arzberger E, Pütz B, Höfler G, Metze D, Sander CA, Rose C, Wolf P, Rütten A, McNiff JM, Kerl H (2000). Primary cutaneous follicle center cell lymphoma with follicular growth pattern. Blood.

[29] Goodlad JR, Krajewski AS, Batstone PJ, McKay P, White JM, Benton EC, Kavanagh GM, Lucraft HH (2002). Primary cutaneous follicular lymphoma—a clinicopathologic and molecular study of 16 cases in support of a distinct entity. Am J Surg Pathol.

[30] Zhou XA, Yang J, Ringbloom KG, Martinez-Escala ME, Stevenson KE, Wenzel AT, Fantini D, Martin HK, Moy AP, Morgan EA, Harkins S, Paxton CN, Hong B, Andersen EF, Guitart J, Weinstock DM, Cerroni L, Choi J, Louissaint A (2021). Genomic landscape of cutaneous follicular lymphomas reveals 2 subgroups with clinically predictive molecular features. Blood Adv.

[31] Kempf W, Kazakov DV, Rutten A, Rupec RA, Talarcik P, Ballova V, Kerl K, Dummer R, Lautenschlager S, Zimmermann DR, Tinguely M (2014). Primary cutaneous follicle center lymphoma with diffuse CD30 expression: a report of 4 cases of a rare variant. J Am Acad Dermatol.

[32] Gángó A, Bátai B, Varga M, Kapczár D, Papp G, Marschalkó M, Kuroli E, Schneider T, Csomor J, Matolcsy A, Bödör C, Szepesi Á (2018). Concomitant 1p36 deletion and TNFRSF14 mutations in primary cutaneous follicle center lymphoma frequently expressing high levels of EZH2 protein. Virchows Archiv : Int J Pathol.

[33] Martin-Guerrero I, Salaverria I, Burkhardt B, Szczepanowski M, Baudis M, Bens S, de Leval L, Garcia-Orad A, Horn H, Lisfeld J, Pellissery S, Klapper W, Oschlies I, Siebert R (2013). Recurrent loss of heterozygosity in 1p36 associated with TNFRSF14 mutations in IRF4 translocation negative pediatric follicular lymphomas. Haematologica.

[34] Nann D, Ramis-Zaldivar JE, Müller I, Gonzalez-Farre B, Schmidt J, Egan C, Salmeron-Villalobos J, Clot G, Mattern S, Otto F, Mankel B, Colomer D, Balagué O, Szablewski V, Lome-Maldonado C, Leoncini L, Dojcinov S, Chott A, Copie-Bergman C, Bonzheim I, Fend F, Jaffe ES, Campo E, Salaverria I, Quintanilla-Martinez L (2020). Follicular lymphoma t(14;18)-negative is genetically a heterogeneous disease. Blood Adv.

[35] Goodlad JR, Krajewski AS, Batstone PJ, McKay P, White JM, Benton EC, Kavanagh GM, Lucraft HH, Scotland, Newcastle Lymphoma G (2003). Primary cutaneous diffuse large B-cell lymphoma—prognostic significance of clinicopathological subtypes. Am J Surg Pathol.

[36] Zinzani PL, Quaglino P, Pimpinelli N, Berti E, Baliva G, Rupoli S, Martelli M, Alaibac M, Borroni G, Chimenti S, Alterini R, Alinari L, Fierro MT, Cappello N, Pileri A, Soligo D, Paulli M, Pileri S, Santucci M, Bernengo MG (2006). Prognostic factors in primary cutaneous B-cell lymphoma: the Italian Study Group for Cutaneous Lymphomas Journal of clinical oncology : official journal of the American Society of. Clin Oncol.

[37] Hoefnagel JJ, Dijkman R, Basso K, Jansen PM, Hallermann C, Willemze R, Tensen CP, Vermeer MH (2005). Distinct types of primary cutaneous large B-cell lymphoma identified by gene expression profiling. Blood.

[38] Schrader AMR, de Groen RAL, Willemze R, Jansen PM, Quint KD, van Wezel T, van Eijk R, Ruano D, Tensen CP, Hauben E, Woei AJF, Busschots AM, van den Berg A, Diepstra A, Vermeer MH, Vermaat JSP (2022). Cell-of-origin classification using the Hans and Lymph2Cx algorithms in primary cutaneous large B-cell lymphomas. Virchows Archiv : Int J Pathol.

[39] Endo M, Ohtsuka M, Watanabe Y, Igari S, Kikuchi N, Taniguchi K, Yoshino T, Yamamoto T (2021). TdT-positive primary cutaneous diffuse large B-cell lymphoma, leg type phenotypically mimicking B-lymphoblastic lymphoma. J Cutan Pathol.

[40] Ronchi A, Zito Marino F, Vitiello P, Caccavale S, Argenziano G, Crisci S, Franco R, Sica A (2021). A case of primary cutaneous B-cell lymphoma with immature features in an old man. Diffuse large B-cell lymphoma with immature features or B-cell lymphoblastic lymphoma?. J Cutan Pathol.

[41] Schrader AMR, Jansen PM, Vermeer MH, Kleiverda JK, Vermaat JSP, Willemze R (2018). High incidence and clinical significance of MYC rearrangements in primary cutaneous diffuse large B-cell lymphoma, leg type. Am J Surg Pathol.

[42] Mareschal S, Pham-Ledard A, Viailly PJ, Dubois S, Bertrand P, Maingonnat C, Fontanilles M, Bohers E, Ruminy P, Tournier I, Courville P, Lenormand B, Duval AB, Andrieu E, Verneuil L, Vergier B, Tilly H, Joly P, Frebourg T, Beylot-Barry M, Merlio JP, Jardin F (2017). Identification of somatic mutations in primary cutaneous diffuse large B-cell lymphoma, leg type by massive parallel sequencing. J Investig Dermatol.

[43] Pham-Ledard A, Beylot-Barry M, Barbe C, Leduc M, Petrella T, Vergier B, Martinez F, Cappellen D, Merlio JP, Grange F (2014). High frequency and clinical prognostic value of MYD88 L265P mutation in primary cutaneous diffuse large B-cell lymphoma, leg-type. JAMA dermatology.

[44] Wright GW, Huang DW, Phelan JD, Coulibaly ZA, Roulland S, Young RM, Wang JQ, Schmitz R, Morin RD, Tang J, Jiang A, Bagaev A, Plotnikova O, Kotlov N, Johnson CA, Wilson WH, Scott DW, Staudt LM (2020). A probabilistic classification tool for genetic subtypes of diffuse large B cell lymphoma with therapeutic implications. Cancer Cell.

[45] Goodlad JR, Krajewski AS, Batstone PJ, McKay P, White JM, Benton EC, Kavanagh GM, Lucraft HH (2003). Primary cutaneous diffuse large B-cell lymphoma: prognostic significance of clinicopathological subtypes. Am J Surg Pathol.

[46] Dummer R, Vermeer MH, Scarisbrick JJ, Kim YH, Stonesifer C, Tensen CP, Geskin LJ, Quaglino P, Ramelyte E (2021). Cutaneous T cell lymphoma Nat Rev Dis Primers.

[47] Tensen CP, Quint KD, Vermeer MH (2022). Genetic and epigenetic insights into cutaneous T-cell lymphoma. Blood.

[48] JR G (2014). The many faces of lymphomatoid papulosis. Diagnostic Histopathology.

[49] Bekkenk MW, Geelen FA, van Voorst Vader PC, Heule F, Geerts ML, van Vloten WA, Meijer CJ, Willemze R (2000). Primary and secondary cutaneous CD30(+) lymphoproliferative disorders: a report from the Dutch Cutaneous Lymphoma Group on the long-term follow-up data of 219 patients and guidelines for diagnosis and treatment. Blood.

[50] Kempf W, Kazakov DV, Scharer L, Rutten A, Mentzel T, Paredes BE, Palmedo G, Panizzon RG, Kutzner H (2013). Angioinvasive lymphomatoid papulosis: a new variant simulating aggressive lymphomas. Am J Surg Pathol.

[51] Kunishige JH, McDonald H, Alvarez G, Johnson M, Prieto V, Duvic M (2009). Lymphomatoid papulosis and associated lymphomas: a retrospective case series of 84 patients. Clin Exp Dermatol.

[52] Benner MF, Willemze R (2009). Applicability and prognostic value of the new TNM classification system in 135 patients with primary cutaneous anaplastic large cell lymphoma. Archives Dermatol.

[53] Beljaards RC, Meijer CJ, Scheffer E, Toonstra J, van Vloten WA, van der Putte SC, Geerts ML, Willemze R (1989). Prognostic significance of CD30 (Ki-1/Ber-H2) expression in primary cutaneous large-cell lymphomas of T-cell origin. A clinicopathologic and immunohistochemical study in 20 patients. Am J Pathol.

[54] Oschlies I KR, Dotlic S, Montes-Moreno S, Ponzoni M, Traverse-Glehen A, Calaminici M, Ferry JA, Ott G, Goodlad JR (2020) The clinico-pathological spectrum of primary cutaneous lymphoma other than mycosis fungoides/Sezary syndrome Virchows Archiv 10.1007/s00428-019-02713-7 31781845

[55] Karai LJ, Kadin ME, Hsi ED, Sluzevich JC, Ketterling RP, Knudson RA, Feldman AL (2013). Chromosomal rearrangements of 6p25.3 define a new subtype of lymphomatoid papulosis. Am J Surg Pathol.

[56] Feldman AL, Law M, Remstein ED, Macon WR, Erickson LA, Grogg KL, Kurtin PJ, Dogan A (2009). Recurrent translocations involving the IRF4 oncogene locus in peripheral T-cell lymphomas. Leukemia.

[57] Wada DA, Law ME, Hsi ED, Dicaudo DJ, Ma L, Lim MS, Souza A, Comfere NI, Weenig RH, Macon WR, Erickson LA, Ozsan N, Ansell SM, Dogan A, Feldman AL (2011). Specificity of IRF4 translocations for primary cutaneous anaplastic large cell lymphoma: a multicenter study of 204 skin biopsies Modern pathology : an official journal of the United States and Canadian Academy of Pathology. Inc.

[58] Onaindia A, Montes-Moreno S, Rodriguez-Pinilla SM, Batlle A, Gonzalez de Villambrosia S, Rodriguez AM, Alegre V, Bermudez GM, Gonzalez-Vela C, Piris MA (2015). Primary cutaneous anaplastic large cell lymphomas with 6p25.3 rearrangement exhibit particular histological features. Histopathology.

[59] Pham-Ledard A, Prochazkova-Carlotti M, Laharanne E, Vergier B, Jouary T, Beylot-Barry M, Merlio JP (2010). IRF4 gene rearrangements define a subgroup of CD30-positive cutaneous T-cell lymphoma: a study of 54 cases. J Invest Dermatol.

[60] Vasmatzis G, Johnson SH, Knudson RA, Ketterling RP, Braggio E, Fonseca R, Viswanatha DS, Law ME, Kip NS, Ozsan N, Grebe SK, Frederick LA, Eckloff BW, Thompson EA, Kadin ME, Milosevic D, Porcher JC, Asmann YW, Smith DI, Kovtun IV, Ansell SM, Dogan A, Feldman AL (2012). Genome-wide analysis reveals recurrent structural abnormalities of TP63 and other p53-related genes in peripheral T-cell lymphomas. Blood.

[61] Oschlies I, Lisfeld J, Lamant L, Nakazawa A, d'Amore ES, Hansson U, Hebeda K, Simonitsch-Klupp I, Maldyk J, Müllauer L, Tinguely M, Stücker M, Ledeley MC, Siebert R, Reiter A, Brugières L, Klapper W, Woessmann W (2013). ALK-positive anaplastic large cell lymphoma limited to the skin: clinical, histopathological and molecular analysis of 6 pediatric cases. A report from the ALCL99 study. Haematologica.

[62] Pulitzer M, Ogunrinade O, Lin O, Steinherz P (2015). ALK-positive (2p23 rearranged) anaplastic large cell lymphoma with localization to the skin in a pediatric patient. J Cutan Pathol.

[63] Velusamy T, Kiel MJ, Sahasrabuddhe AA, Rolland D, Dixon CA, Bailey NG, Betz BL, Brown NA, Hristov AC, Wilcox RA, Miranda RN, Medeiros LJ, Jeon YK, Inamdar KV, Lim MS, Elenitoba-Johnson KS (2014). A novel recurrent NPM1-TYK2 gene fusion in cutaneous CD30-positive lymphoproliferative disorders. Blood.

[64] Crescenzo R, Abate F, Lasorsa E, Tabbo F, Gaudiano M, Chiesa N, Di Giacomo F, Spaccarotella E, Barbarossa L, Ercole E, Todaro M, Boi M, Acquaviva A, Ficarra E, Novero D, Rinaldi A, Tousseyn T, Rosenwald A, Kenner L, Cerroni L, Tzankov A, Ponzoni M, Paulli M, Weisenburger D, Chan WC, Iqbal J, Piris MA, Zamo A, Ciardullo C, Rossi D, Gaidano G, Pileri S, Tiacci E, Falini B, Shultz LD, Mevellec L, Vialard JE, Piva R, Bertoni F, Rabadan R, Inghirami G (2015). Convergent mutations and kinase fusions lead to oncogenic STAT3 activation in anaplastic large cell lymphoma. Cancer Cell.

[65] Prieto-Torres L, Rodriguez-Pinilla SM, Onaindia A, Ara M, Requena L, Piris M (2019). CD30-positive primary cutaneous lymphoproliferative disorders: molecular alterations and targeted therapies. Haematologica.

[66] Sun J, Yi S, Qiu L, Fu W, Wang A, Liu F, Wang L, Wang T, Chen H, Wang L, Kadin ME, Tu P, Wang Y (2018). J Invest Dermatol.

[67] Chott A, Vonderheid EC, Olbricht S, Miao NN, Balk SP, Kadin ME (1996). The dominant T cell clone is present in multiple regressing skin lesions and associated T cell lymphomas of patients with lymphomatoid papulosis. J Invest Dermatol.

[68] de la Garza Bravo MM, Patel KP, Loghavi S, Curry JL, Torres Cabala CA, Cason RC, Gangar P, Prieto VG, Medeiros LJ, Duvic M, Tetzlaff MT (2015). Shared clonality in distinctive lesions of lymphomatoid papulosis and mycosis fungoides occurring in the same patients suggests a common origin. Hum Pathol.

[69] Xerri L, Adelaide J, Avenin M, Guille A, Taix S, Bonnet N, Carbuccia N, Garnier S, Mescam L, Murati A, Chaffanet M, Coso D, Bouabdallah R, Bertucci F, Birnbaum D (2019). Common origin of sequential cutaneous CD30+ lymphoproliferations with nodal involvement evidenced by genome-wide clonal evolution. Histopathology.

[70] Beltraminelli H, Leinweber B, Kerl H, Cerroni L (2009). Primary cutaneous CD4+ small-/medium-sized pleomorphic T-cell lymphoma: a cutaneous nodular proliferation of pleomorphic T lymphocytes of undetermined significance? A study of 136 cases. Am J Dermatopathol.

[71] Garcia-Herrera A, Colomo L, Camos M, Carreras J, Balague O, Martinez A, Lopez-Guillermo A, Estrach T, Campo E (2008). Primary cutaneous small/medium CD4+ T-cell lymphomas: a heterogeneous group of tumors with different clinicopathologic features and outcome. J Clin Oncol : Off J Am Soc Clin Oncol.

[72] Grogg KL, Jung S, Erickson LA, McClure RF, Dogan A (2008). Primary cutaneous CD4-positive small/medium-sized pleomorphic T-cell lymphoma: a clonal T-cell lymphoproliferative disorder with indolent behavior Modern pathology : an official journal of the United States and Canadian Academy of Pathology. Inc.

[73] Leinweber B, Beltraminelli H, Kerl H, Cerroni L (2009). Solitary small- to medium-sized pleomorphic T-cell nodules of undetermined significance: clinical, histopathological, immunohistochemical and molecular analysis of 26 cases. Dermatol (Basel, Switzerland).

[74] Rodríguez-Pinilla SM, Atienza L, Murillo C, Pérez-Rodríguez A, Montes-Moreno S, Roncador G, Pérez-Seoane C, Domínguez P, Camacho FI, Piris MA (2008). Peripheral T-cell lymphoma with follicular T-cell markers. Am J Surg Pathol.

[75] Baum CL, Link BK, Neppalli VT, Swick BL, Liu V (2011). Reappraisal of the provisional entity primary cutaneous CD4+ small/medium pleomorphic T-cell lymphoma: a series of 10 adult and pediatric patients and review of the literature. J Am Acad Dermatol.

[76] Agnarsson BA, Vonderheid EC, Kadin ME (1990). Cutaneous T cell lymphoma with suppressor/cytotoxic (CD8) phenotype: identification of rapidly progressive and chronic subtypes. J Am Acad Dermatol.

[77] Berti E, Tomasini D, Vermeer MH, Meijer CJ, Alessi E, Willemze R (1999). Primary cutaneous CD8-positive epidermotropic cytotoxic T cell lymphomas A distinct clinicopathological entity with an aggressive clinical behavior. Am J Pathol.

[78] Robson A, Assaf C, Bagot M, Burg G, Calonje E, Castillo C, Cerroni L, Chimenti N, Dechelotte P, Franck F, Geerts M, Gellrich S, Goodlad J, Kempf W, Knobler R, Massone C, Meijer C, Ortiz P, Petrella T, Pimpinelli N, Roewert J, Russell-Jones R, Santucci M, Steinhoff M, Sterry W, Wechsler J, Whittaker S, Willemze R, Berti E (2015). Aggressive epidermotropic cutaneous CD8+ lymphoma: a cutaneous lymphoma with distinct clinical and pathological features Report of an EORTC Cutaneous Lymphoma Task Force Workshop. Histopathology.

[79] Robson A, Assaf C, Bagot M, Burg G, Calonje E, Castillo C, Cerroni L, Chimenti N, Dechelotte P, Franck F, Geerts M, Gellrich S, Goodlad J, Kempf W, Knobler R, Massone C, Meijer C, Ortiz P, Petrella T, Pimpinelli N, Roewert J, Russell-Jones R, Santucci M, Steinhoff M, Sterry W, Wechsler J, Whittaker S, Willemze R, Berti E (2015). Aggressive epidermotropic cutaneous CD8(+) lymphoma: a cutaneous lymphoma with distinct clinical and pathological features Report of an EORTC Cutaneous Lymphoma Task Force Workshop. Histopathology.

[80] Bastidas Torres AN, Cats D, Out-Luiting JJ, Fanoni D, Mei H, Venegoni L, Willemze R, Vermeer MH, Berti E, Tensen CP (2022). Deregulation of JAK2 signaling underlies primary cutaneous CD8(+) aggressive epidermotropic cytotoxic T-cell lymphoma. Haematologica.

[81] Greenblatt D, Ally M, Child F, Scarisbrick J, Whittaker S, Morris S, Calonje E, Petrella T, Robson A (2013). Indolent CD8(+) lymphoid proliferation of acral sites: a clinicopathologic study of six patients with some atypical features. J Cutan Pathol.

[82] Petrella T, Maubec E, Cornillet-Lefebvre P, Willemze R, Pluot M, Durlach A, Marinho E, Benhamou JL, Jansen P, Robson A, Grange F (2007). Indolent CD8-positive lymphoid proliferation of the ear: a distinct primary cutaneous T-cell lymphoma?. Am J Surg Pathol.

[83] Suchak R, O'Connor S, McNamara C, Robson A (2010). Indolent CD8-positive lymphoid proliferation on the face: part of the spectrum of primary cutaneous small-/medium-sized pleomorphic T-cell lymphoma or a distinct entity?. J Cutan Pathol.

[84] Gaulard P. BE, Willemze R., Petrella T., Jaffe E.S. (2017) Primary cutaneous peripheral T-cell lymphoma, rare subtypes. In: Swerdlow S.H. CE, Harris N.L., Jaffe E.S., Pileri S.A., Stein H., Thiele J., Vardiman J.W. (ed) WHO classification of tumours of haematopoietic and lymphoid tissues, Revised 4th Edition.edn. IARC Press, Lyon, pp. 397–402

[85] Beltraminelli H, Mullegger R, Cerroni L (2010). Indolent CD8+ lymphoid proliferation of the ear: a phenotypic variant of the small-medium pleomorphic cutaneous T-cell lymphoma?. J Cutan Pathol.

[86] Kempf W, Petrella T, Willemze R, Jansen P, Berti E, Santucci M, Geissinger E, Cerroni L, Maubec E, Battistella M, Goodlad J, Guenova E, Lappalainen K, Ranki A, Craig P, Calonje E, Martin B, Whittaker S, Oschlies I, Wehkamp U, Nicolay JP, Wobser M, Scarisbruck J, Pimpinelli N, Stadler R, Kerl French K, Quaglino P, Lin J, Chen L, Beer M, Emanuel P, Dalle S, Robson A (2022). Clinical, histopathological and prognostic features of primary cutaneous acral CD8(+) T-cell lymphoma and other dermal CD8(+) cutaneous lymphoproliferations: results of an EORTC Cutaneous Lymphoma Group workshop. Br J Dermatol.

[87] Wobser M, Roth S, Reinartz T, Rosenwald A, Goebeler M, Geissinger E (2015). CD68 expression is a discriminative feature of indolent cutaneous CD8-positive lymphoid proliferation and distinguishes this lymphoma subtype from other CD8-positive cutaneous lymphomas. Br J Dermatol.

[88] Jaffe E.S. GP, Cerroni L. (2017) Subcutaneous panniculitis-like T-cell lymphoma. In: Swerdlow S.H. CE, Harris N.L., Jaffe E.S., Pileri S.A., Stein H., Thiele J., Vardiman J.W. (ed) WHO Classification of Tumours of Haematopoietic and Lymphoid Tissues, Revised 4th Edition.edn. IARC Press, Lyon, pp. 383–385

[89] Oschlies I, Simonitsch-Klupp I, Maldyk J, Konovalov D, Abramov D, Myakova N, Lisfeld J, Attarbaschi A, Kontny U, Woessmann W, Klapper W (2015). Subcutaneous panniculitis-like T-cell lymphoma in children: a detailed clinicopathological description of 11 multifocal cases with a high frequency of haemophagocytic syndrome. Br J Dermatol.

[90] Willemze R, Jansen PM, Cerroni L, Berti E, Santucci M, Assaf C, Canninga-van Dijk MR, Carlotti A, Geerts ML, Hahtola S, Hummel M, Jeskanen L, Kempf W, Massone C, Ortiz-Romero PL, Paulli M, Petrella T, Ranki A, Peralto JL, Robson A, Senff NJ, Vermeer MH, Wechsler J, Whittaker S, Meijer CJ (2008). Subcutaneous panniculitis-like T-cell lymphoma: definition, classification, and prognostic factors: an EORTC Cutaneous Lymphoma Group Study of 83 cases. Blood.

[91] Bosisio F, Boi S, Caputo V, Chiarelli C, Oliver F, Ricci R, Cerroni L (2015). Lobular panniculitic infiltrates with overlapping histopathologic features of lupus panniculitis (lupus profundus) and subcutaneous T-cell lymphoma: a conceptual and practical dilemma. Am J Surg Pathol.

[92] Gayden T, Sepulveda FE, Khuong-Quang DA, Pratt J, Valera ET, Garrigue A, Kelso S, Sicheri F, Mikael LG, Hamel N, Bajic A, Dali R, Deshmukh S, Dervovic D, Schramek D, Guerin F, Taipale M, Nikbakht H, Majewski J, Moshous D, Charlebois J, Abish S, Bole-Feysot C, Nitschke P, Bader-Meunier B, Mitchell D, Thieblemont C, Battistella M, Gravel S, Nguyen VH, Conyers R, Diana JS, McCormack C, Prince HM, Besnard M, Blanche S, Ekert PG, Fraitag S, Foulkes WD, Fischer A, Neven B, Michonneau D, de Saint Basile G, Jabado N (2018). Germline HAVCR2 mutations altering TIM-3 characterize subcutaneous panniculitis-like T cell lymphomas with hemophagocytic lymphohistiocytic syndrome. Nat Genet.

[93] Koh J, Jang I, Mun S, Lee C, Cha HJ, Oh YH, Kim JM, Han JH, Paik JH, Cho J, Ko YH, Park CS, Go H, Huh J, Kim K, Jeon YK (2021). Genetic profiles of subcutaneous panniculitis-like T-cell lymphoma and clinicopathological impact of HAVCR2 mutations. Blood Adv.

[94] Sonigo G, Battistella M, Beylot-Barry M, Ingen-Housz-Oro S, Franck N, Barete S, Boulinguez S, Dereure O, Bonnet N, Socié G, Brice P, Boccara O, Bodemer C, Adamski H, D'Incan M, Ortonne N, Fraitag S, Brunet-Possenti F, Dalle S, Suarez F, Marçais A, Skowron F, Haidar D, Maubec E, Bohelay G, Laroche L, Mahé A, Birckel E, Bouaziz JD, Brocheriou I, Dubois R, Faiz S, Fadlallah J, Ram-Wolff C, Carlotti A, Bens G, Balme B, Vergier B, Laurent-Roussel S, Deschamps L, Carpentier O, Moguelet P, Herve G, Comoz F, Le Gall F, Leverger G, Finon A, Augereau O, Bléchet C, Kerdraon R, Lamant L, Tournier E, Franck F, Costes-Martineau V, Szablewski V, Taix S, Beschet I, Guerin F, Sepulveda FE, Bagot M, de Saint Basile G, Michonneau D, de Masson A (2020). HAVCR2 mutations are associated with severe hemophagocytic syndrome in subcutaneous panniculitis-like T-cell lymphoma. Blood.

[95] Wolf Y, Anderson AC, Kuchroo VK (2020). TIM3 comes of age as an inhibitory receptor. Nat Rev Immunol.

[96] Guitart J, Weisenburger DD, Subtil A, Kim E, Wood G, Duvic M, Olsen E, Junkins-Hopkins J, Rosen S, Sundram U, Ivan D, Selim MA, Pincus L, Deonizio JM, Kwasny M, Kim YH (2012). Cutaneous gammadelta T-cell lymphomas: a spectrum of presentations with overlap with other cytotoxic lymphomas. Am J Surg Pathol.

[97] Toro JR, Liewehr DJ, Pabby N, Sorbara L, Raffeld M, Steinberg SM, Jaffe ES (2003). Gamma-delta T-cell phenotype is associated with significantly decreased survival in cutaneous T-cell lymphoma. Blood.

[98] Toro JR, Beaty M, Sorbara L, Turner ML, White J, Kingma DW, Raffeld M, Jaffe ES (2000). gamma delta T-cell lymphoma of the skin: a clinical, microscopic, and molecular study. Arch Dermatol.

[99] Daniels J, Doukas PG, Escala MEM, Ringbloom KG, Shih DJH, Yang J, Tegtmeyer K, Park J, Thomas JJ, Selli ME, Altunbulakli C, Gowthaman R, Mo SH, Jothishankar B, Pease DR, Pro B, Abdulla FR, Shea C, Sahni N, Gru AA, Pierce BG, Louissaint A, Guitart J, Choi J (2020). Cellular origins and genetic landscape of cutaneous gamma delta T cell lymphomas. Nature Commun.

[100] Battistella M, Beylot-Barry M, Bachelez H, Rivet J, Vergier B, Bagot M (2012). Primary cutaneous follicular helper T-cell lymphoma: a new subtype of cutaneous T-cell lymphoma reported in a series of 5 cases. Archives Dermatol.

[101] Bekkenk MW, Vermeer MH, Jansen PM, van Marion AM, Canninga-van Dijk MR, Kluin PM, Geerts ML, Meijer CJ, Willemze R (2003). Peripheral T-cell lymphomas unspecified presenting in the skin: analysis of prognostic factors in a group of 82 patients. Blood.

